# High Mobility Group B Proteins, Their Partners, and Other Redox Sensors in Ovarian and Prostate Cancer

**DOI:** 10.1155/2016/5845061

**Published:** 2015-11-23

**Authors:** Aida Barreiro-Alonso, Mónica Lamas-Maceiras, Esther Rodríguez-Belmonte, Ángel Vizoso-Vázquez, María Quindós, M. Esperanza Cerdán

**Affiliations:** ^1^Center for Investigacións Científicas Avanzadas (CICA), Department of Cellular and Molecular Biology, Sciences Faculty, University of Coruña, 15071 A Coruña, Spain; ^2^Translational Cancer Research Group, A Coruña Biomedical Research Institute (INIBIC), Carretera del Pasaje s/n, 15006 A Coruña, Spain

## Abstract

Cancer cells try to avoid the overproduction of reactive oxygen species by metabolic rearrangements. These cells also develop specific strategies to increase ROS resistance and to express the enzymatic activities necessary for ROS detoxification. Oxidative stress produces DNA damage and also induces responses, which could help the cell to restore the initial equilibrium. But if this is not possible, oxidative stress finally activates signals that will lead to cell death. High mobility group B (HMGB) proteins have been previously related to the onset and progressions of cancers of different origins. The protein HMGB1 behaves as a redox sensor and its structural changes, which are conditioned by the oxidative environment, are associated with different functions of the protein. This review describes recent advances in the role of human HMGB proteins and other proteins interacting with them, in cancerous processes related to oxidative stress, with special reference to ovarian and prostate cancer. Their participation in the molecular mechanisms of resistance to cisplatin, a drug commonly used in chemotherapy, is also revised.

## 1. Introduction

Reactive oxygen species (ROS), generated as consequence of oxidative metabolism, activate signal transduction pathways, which contribute to cellular homeostasis [1]. Metabolically active cells, neutrophils, and macrophages from the immune system produce high levels of ROS. Consequently, the recruitment of immune cells during chronic inflammation increases oxidative stress (OS) in the microenvironment [2]. Exogenous sources, such as cigarette smoke and UV-light, also contribute to increasing the total cellular ROS content. The maintenance of the steady-state equilibrium between ROS generation and elimination is crucial for cell survival, while its loss causes cell death by different mechanisms triggered by oxidative damage. Cancer cells demand high energy production to sustain their pathological increase in proliferation rate. Thus, in order to avoid excessive ROS generation, they switch the utilization of metabolic pathways that require mitochondrial respiration to fermentation [3]. They also develop specific strategies to increase ROS resistance, which include deviation of the glycolytic flux into the pentose phosphate pathway (PPP) or changes in other enzymatic mechanisms enhancing ROS detoxification [3, 4]. In cancer cells, ROS production is mainly due to overexpression of the NADPH oxidase [3]. Paradoxically, the antioxidant enzymes necessary for ROS elimination use the NADPH coenzyme; therefore, the PPP is important as a source of NADPH reducing power [3]. While a balance between enhanced ROS production and detoxification can be maintained, cancer cells will proliferate and survive. Commonly used radio- and chemotherapies are prooxidant strategies that alter cancer cells through ROS modulation and induce cell death [5, 6].

Changes in the redox state of cells affect proteins, lipids, and nucleic acids in different ways. HMGB1 is an abundant protein, 10^6^ molecules per cell [7], which has been postulated as a redox sensor [8]. HMGB1 is also related to the hallmarks of cancer as described by Hanahan and Weinberg [9]. These are as follows: sustained proliferative signalling, cell death resistance, replicative immortality, genome instability and increased mutations, tumour-promoted inflammation, insensibility to growth repressors, deregulation of cellular energetics, evasion of immune destruction, induction of metastasis, and promotion of angiogenesis. The biological functions of HMGB1 are diverse in normal cells and during the start and progression of cancer. Remarkably, these functions change depending on its redox state and cellular compartment. In the nucleus it behaves as a DNA chaperone, sustains nucleosome dynamics and chromosomal stability, and contributes to telomere maintenance [10]. It also modulates gene transcription and recombination [7]. Besides, HMGB1 participates in DNA repair by different mechanisms such as nucleotide excision repair, NER, mismatch repair, MMR, base excision repair, BER, and double strand break repair, DSBR [11]. In the cytoplasm, HMGB1 binds the protein beclin1, increases autophagy, inhibits apoptosis, and regulates mitochondrial morphology and function [12]. HMGB1 can be secreted by activated macrophages, monocytes, natural killer cells, and dendritic cells or can be released from necrotic or injured cells mainly during oxidative stress [13, 14]. Once HMGB1 becomes an extracellular signal, it binds to several cell surface receptors, principally to the receptor for advanced glycation end products (RAGE) and toll-like receptors (TLRs) and activates nuclear factor kappa B (NF-*κ*B) signalling [15] and other downstream signalling pathways [12]. As a result, HMGB1 modulates immune and inflammatory responses and promotes cell proliferation, angiogenesis, and cell adhesion and migration. Curiously, oncogenic and tumour-suppressive activities have been assigned to HMGB1 at different stages of tumour genesis and therapy [12]. Moreover, it has been reported that Tax1, an oncogenic protein of viral origin, upregulates HMGB1 levels, which suggests that cancers of viral origin could also be related to HMGB1 deregulation [16].

Different isoforms of the human protein HMGB1, encoded by the* HMGB1* gene, have been reported [17] and other genes (*HMGB2* and* HMGB3*—alias HMG2a—and* HMGB4*), encoding similar although less studied HMGB proteins, are present in the human genome [18–20]. This review describes recent advances in the biological functions of human HMGB proteins and other proteins interacting with them, in cancerous processes related to OS, with special reference to ovarian and prostate cancer. These two malignancies have been previously related to redox imbalance and deregulation of the nuclear factor erythroid 2-related factor 2 gene,* NRF2*, encoding a transcription factor that binds to antioxidant response elements (AREs) and that is regarded as a promising therapeutic target [21, 22]. The molecular mechanisms of resistance to cisplatin, commonly used in chemotherapy of ovarian and prostate cancers, and their interplay with HMGB proteins are also reviewed.

## 2. HMGB Proteins as Redox Sensors

**Figure 1:**
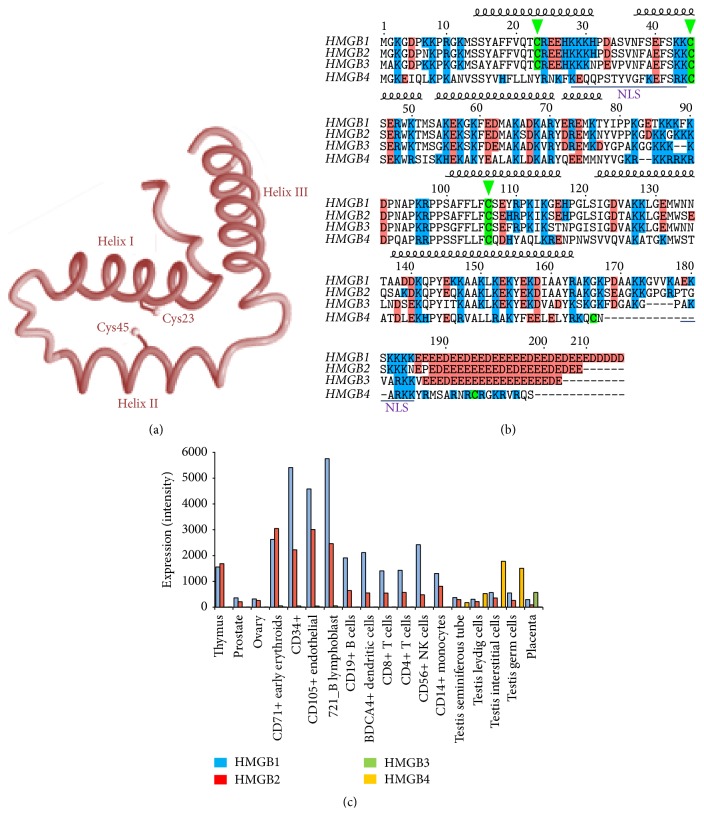
Comparative structure and expression of human HMGB proteins. (a) The HMG box folding characteristic of HMGB proteins showing the two Cys that form the disulphide bond. (b) CLUSTAL alignment of the human HMGB proteins. The three alpha-helix-stretches of HMG box-A and Box-B are indicated by their secondary structure; the acidic tail in the carboxylic end is signalled in red; cysteines are in green; the two NLSs characterized in HMGB1 are underlined in yellow. (c) Levels of expression of mRNAs from HMGB proteins are according to data from BioGPS (http://BioGPS.org).

HMGB1 is so far the most studied member among the human HMGB protein family. It has many different functions that depend on its redox state and posttranscriptional modifications, like acetylation, which determine its cellular or extracellular localization. HMGB1 is polyacetylated near its nuclear-localization sequences (NLSs) and this modification blocks the interaction with the nuclear importer [23]. Acetylated cytosolic HMGB1 is incorporated into cytoplasmic secretory vesicles that allow the regulated secretion of the protein [24]. The four human HMGB proteins have two positively charged DNA binding domains, HMG A-box and HMG B-box, folded in the characteristic L-shaped architecture (Figure 1(a)). Each domain is formed by three alpha-helix-stretches which are indicated in Figure 1(b). In HMGB1, the HMG A-box includes amino acids 1–79, and the HMG B-box is formed by amino acids 89–163. The acidic carboxyl terminus, amino acids 186–215, is negatively charged and has an extended and flexible structure, which interacts with residues within and between the two HMG boxes [25] although it has the highest affinity for the HMG B-box [26]. Many of the redox changes, associated with different functions of HMGB1, are conditioned by the environment and, therefore, HMGB1 is considered a master redox sensor. This function depends on three cysteine residues at positions 23, 45, and 106, which can be in reduced state, as thiols, or oxidized, as disulphide bonds. In moderate oxidative conditions, Cys23 and Cys45 easily form an intramolecular disulphide bridge, while Cys106 remains reduced (the semioxidized HMGB1 form). Nuclear magnetic resonance (NMR) spectroscopy studies of HMG A-box have shown that the redox potential of the Cys23-Cys45 pair is within the physiological intracellular range [8]. The formation of the disulphide bond is favoured with a standard redox potential as low as −237 mV [27]; in consequence, a significant fraction of HMGB1 is expected to be in the semioxidized form within cells [8]. This Cys-Cys bond is a target of glutathione-dependent reduction by glutaredoxin [28]. The proximity of these Cys residues to amino acids that are necessary for DNA binding [29] explains the importance of redox-regulated conformational changes in HMGB1, which may modulate their affinity for DNA. Redox changes may also affect the interaction with other proteins and receptors and modify their biological functions. Cysteines can be further oxidized to sulfenic (RSOH), sulfinic (RSO_2_H), or sulfonic (RSO_3_H) acids under increased OS pressure [28].

## 3. Structural and Functional Similarities and Differences between Human HMGBs

The tertiary structure of HMGB1 A-box [29] reveals that Cys23 and Cys45 are located at the centre of helix I and helix II, respectively, opposing each other and at a distance that allows the formation of a disulphide bond under appropriate oxidative conditions (Figure 1(a)). The proteins HMGB1, HMGB2, HMGB3, and HMGB4 share a great similarity in their amino acid sequences as shown in the CLUSTALW alignment (Figure 1(b)). Only HMGB4 has some remarkable differences with the others, but even so, it conserves high similarity. Cys23 and Cys45 are conserved in HMGB2 and HMGB3. Cys23 is absent in HMGB4, and certainly the DNA binding capacity of HMGB4 is independent of redox changes [30].

Cys106 is involved in the nuclear localization of HMGB1 [28] and this residue is conserved in the four human HMGB proteins (Figure 1(b)). Two nuclear location signals (NLSs), which are rich in lysine residues and extend from amino acids 28–44 and 179–185, respectively, have been described in HMGB1 [31]. The NLSs are well conserved as shown in the alignment (Figure 1(b)) although in HMGB4 they show more variation. Although Cys106 is not present inside the NLS, thiols may participate in nuclear transport by a number of indirect mechanisms such as nuclear pore complex binding [32], ubiquitination [33], or transporter interaction [34, 35]. Consequently, Cys106 conservation may be important to preserve the nuclear functions of these proteins.

Besides the absence of Cys23, the two most outstanding features of HMGB4, in comparison to the other HMGB proteins, are the presence of two additional cysteines at positions 164 and 178 and the absence of the acidic tail in the carboxylic end (Figure 1(b)). To our knowledge the biological significance of Cys164 and Cys168 in HMGB4 has not been studied yet. The function of the acidic tail in HMGB proteins is related to the interaction with other proteins, like nucleosome core histone H3 [36], and also to the stabilization of specific HMGB folding forms, because in HMGB1 it can interact with basic residues present in HMG B-box or in the interconnection of the two HMG boxes [25, 26].

HMGB proteins are widely expressed, except in cells without nucleus [37]. Data from microarrays reveal that* HMGB1* and* HMGB2* genes are the highest expressed in immune cells.* HMGB3* expression is relatively high in placenta and* HMGB4* expression is specific of testis (Figure 1(c)). The functional significance of these variations is unknown, since specific studies have not been reported. One possible explanation is that they may have different functions in different tissues, which may be associated with binding to tissue-specific protein partners. Remarkably, abnormal mRNA and protein levels of these proteins have been detected in numerous cancers, including ovarian and prostate [38–41].

## 4. HMGB Interactions with Nuclear Proteins

After ribosomal synthesis, HMGB1 is imported into the nucleus where it interacts with the minor groove of free DNA through the HMG boxes [42] and it behaves as a DNA chaperone [43]. HMGB1 also binds to packed DNA, relaxes the structure of nucleosomes, promotes their sliding, and relaxes chromatin; thus, by its ability to bend DNA, HMGB1 favours the accessibility of other proteins to chromatin [44]. The C-terminal unstructured acidic tail of HMGB1 interacts with the N-terminal unstructured tail of histone H3, which is close to the DNA entry/exit points around the nucleosome dyad, thus positioning HMGB1 on the linker DNA [36]. This DNA chaperone function would explain the implication of HMGB proteins in wide variety of nuclear processes such as DNA replication, recombination, transcription, telomere maintenance, and diverse mechanisms of DNA repair [45–47]. OS causes DNA damage and it also affects proteins involved in these DNA-related processes. The OS induced responses could help the cell to restore the initial equilibrium or if the feedback to the initial status is not possible, they could activate pathways that would lead to cell death.

**Figure 2:**
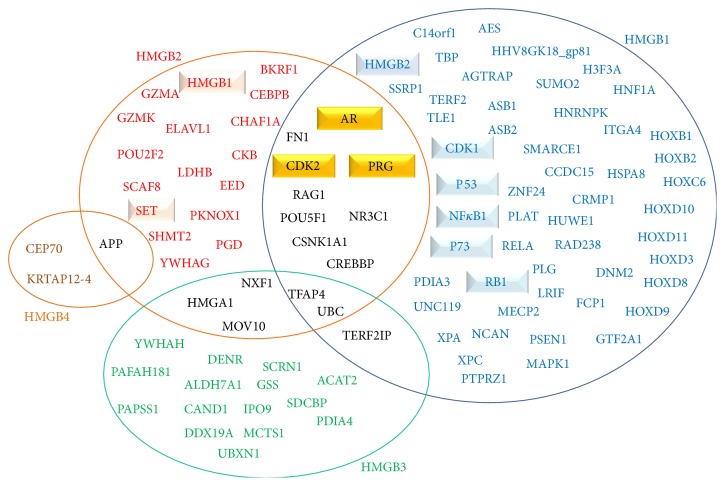
Venn diagram of HMGB protein interaction partners. Those reported as modified during ovarian or prostate cancer progression are highlighted inside the boxes. The figure has been done considering the public results from BioGRID (http://thebiogrid.org/, as available on date 05/31/2015).

Several proteins have been recognised as HMGB1, HMGB2, HMGB3, or HMGB4-interactants by diverse approaches and results are deposited in BioGRID (http://thebiogrid.org/). A summary of these interactions is presented by a Venn diagram (Figure 2). The results in BioGRID include more interactions detected for HMGB1 or HMGB2 than for HMGB3 or HMGB4 proteins, a feature that will probably change with the progression of ongoing interactome projects in the near future. HMGB1 and HMGB2 interact with each other and they have common interactors like the nuclear hormonal receptors which are deregulated in prostate and ovarian cancers [48–50]. The functions of the HMGB partners as well as their sensibility to OS could help us to understand the role of HMGB proteins in the response to oxidative damage and their implications in the origin and progression of cancer.

In the nucleus, HMGB proteins interact with a number of transcription factors, among them tumour suppressors like P53 [51–53] or its homolog P73 [54]. It has been reported that nuclear retention of HMGB1 and P53 depends on the formation of a complex between them and, without their binding partner, HMGB1 or P53 can return more easily to the cytoplasm [55]. The interaction with P53 is of particular importance in the relation of HMGB1 with OS and cancer since P53 also functions as a redox sensor in the cell [56]. It has been recently reported that P53 can directly sense OS through DNA-mediated charge transport and that purine regions with lower redox potential facilitate higher P53-DNA dissociation [57]. The association* in vivo* and* in vitro* of each of the four HMGB proteins with the retinoblastoma protein (RB) occurs through a common LXCXE/D motif that is necessary for modulation of cancer cell growth [58, 59].

HMGB1 interacts differentially with members of the REL family of transcription factors (RELA/P65, c-REL, RELB, P50/NF-*κ*B1, and P52/NF-*κ*B2) like NF-*κ*B1 [60]. In the nucleus NF-*κ*B1 promotes cell proliferation and antiapoptosis by transcriptional regulation, playing a key role in tumour genesis and progression [61]. HMGB1 and HMGB2 interact with nuclear steroid hormone receptors including estrogen, androgen, and glucocorticoid receptors [48–50] favouring the binding to their DNA targets [62, 63]. The interactions with hormone receptors are of relevance taking into account the hormonal dependence of several cancers [40].

HMGB1 binds to cyclin-dependent kinases like CDK2 that control transcriptional regulation of genes related to cell cycle progression [64]. HMGB1 also interacts with topoisomerase II alpha, highly expressed in tumours and involved in replication and chromosomal segregation and recombination, and stimulates its catalytic activity [47]. In absence of RB, HMGB1 and HMGB2 modulate the binding of the transcription factor NF-Y to the topoisomerase II alpha promoter [65]. NF-Y recognizes CCAAT boxes and has been related to different types of cancer [66].

The high mobility group A (HMGA) proteins belong, as HMGB proteins, to the HMG family and are characterized by the “AT hook” domain for DNA binding, instead of the HMG box present in HMGB proteins. The HMGA proteins alter chromatin structure and thereby regulate the transcription of several genes, being also implicated in the development of benign and malignant neoplasms [67]. HMGA proteins have been related to the process by which epithelial cells change to mesenchymal type (the epithelial-to-mesenchymal transition, or EMT). During EMT, epithelial cells lose their cell polarity and cell-cell adhesion capacity, which leads to constriction caused by the two vicinal cells and extrusion of a new mesenchymal cell. This stromal mesenchymal cell has both migratory and invasive capacities and also has the potential to differentiate into a variety of cell types. EMT is essential for numerous developmental processes and also occurs in the initiation of metastasis, being very important in tumours of epithelial origin. Carcinoma cells in the primary tumour lose cell-cell adhesion mediated by E-cadherin and gain access to the bloodstream through extravasation [68]. HMGA2, once induced by transforming growth factor *β* (TGF*β*), associates with SMAD complexes and induces expression of the SNAIL transcription factors, which controls epithelial-mesenchymal transitions by repressing E-cadherin [69] and Twist [70] expression. No direct effect of the HMGB proteins in this process has been described and although HMGB1, HMGB2, and HMGB3 interact with HMGA1 [71], a direct interaction with HMGA2 has not been reported. The study of HMGB-HMGA interactions is an interesting area to explore in relation to EMT.

HMGB proteins are able to bind to other nuclear proteins that do not have DNA binding capacity but that have a role in modifying transcription and in the onset of cancer. HMGB1 interacts with the aminoterminal enhancer of split, AES, [72], which plays an important role in tumour metastasis by regulating cell adhesion through changes in RND3 expression [73]. RND3 (alias RHOE) is a member of the small GTPases and regulates actin cytoskeleton organization and cell migration [74], as well as proliferation, differentiation, and apoptosis [75–77].

Besides the effects caused by direct interactions between HMGB proteins and other regulators of gene expression, we also have to consider the cross-regulation that operates to modulate the expression of all these factors. In this sense, it has been recently shown that the enhanced ectopic expression of HMGB1 decreases BCL-2-like protein 4 (BAX) and P53 expression, while it enhances B-cell lymphoma extra large (BCL-XL), B-cell lymphoma 2 (BCL-2), cyclin D1, and NF-*κ*B expression [78]. This causes activation of cell growth and diminishes cell death [78].

## 5. HMGB Proteins in Survival versus Cell Death Control

Redox imbalance in the cells could lead to oxidative damage in the nucleus, with the consequent genome instability, and other processes related to malignancy along cancer origin and propagation. The progression of the OS response in the cell is accompanied by changes that might affect cell survival, providing reparative mechanisms, or promoting cell death. However, death or survival of a single cell could be good or bad for the organism. If survival affects a cell without a serious compromise in genome integrity and stability, the tissue will probably restore its healthy status. But if a cell with previous hallmarks for cancerous progression survives, its success is paradoxically detrimental for the tissue and the organism.

**Figure 3:**
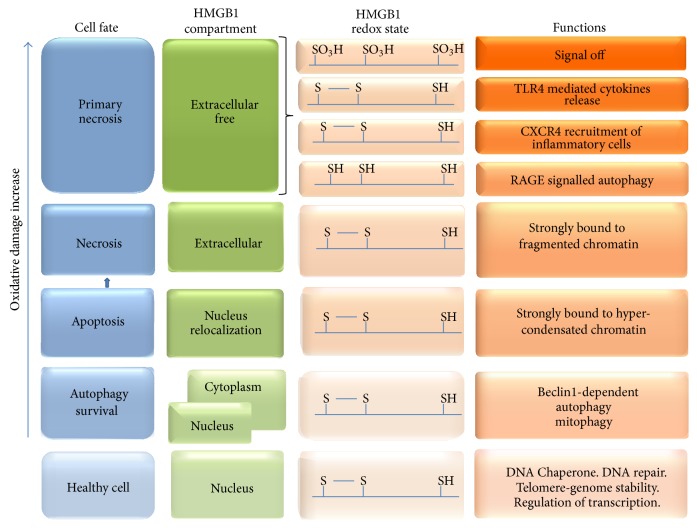
Relationship between oxidative stress progression, cell fate, HMGB1 redox states, and HMGB1 functions.

Cell death can occur by different mechanisms and the oxidative state of the cell and its microenvironment is a key determinant for their selection (Figure 3). When oxidative damage starts and ROS production is enough for starting the mitochondrial permeability transition (MPT), mitophagy and autophagy allow the cell to recycle damaged elements and survive. Autophagy and its selective form mitophagy that destroys damaged mitochondria require the formation and progression of the phagophore to finally produce the autophagosome. Then, the autophagosome fuses to the lysosome to constitute the autolysosome, where the degradation of the sequestered elements occurs [79]. If the oxidative damage persists, the integrity of the mitochondria is affected and cytochrome *c* is released; this molecule signals apoptosis and, consequently, cell death without immunogenic activation. Finally, with the highest levels of oxidative damage, necrosis is established and with it the possibility of a wide immunogenic activation [80].

As shown in Figure 3, in viable cells, HMGB1 is mostly localized in the nucleus associated with DNA and proteins in chromatin. Low acetylation of histones, observed during apoptosis, causes a hypercondensation of chromatin and the irreversible HMGB1 binding; this binding is a canonical characteristic of alarmins like HMGB1 [80]. If the apoptotic cell is not cleared by macrophages, secondary necrosis is produced and the instability of cellular membranes allows HMGB1 to be released to the extracellular media strongly bound to DNA [37]. If necrosis is primary, not derived from previously apoptotic cells, HMGB1 release is also observed, but in this case the protein is free, not associated with DNA [81]. ATP depletion mediated by poly[ADP-ribose] polymerase 1 (PARP1) also regulates HMGB1 release during necrosis [82].

HMGB1 has important functions controlling the balance between autophagy and apoptosis. In the nucleus, as a regulator of transcription, and in the cytoplasm, by binding to regulator proteins, HMGB1 controls these processes. Under OS or other types of stress, hyperacetylation of NLSs promotes HMGB1 translocation from the nucleus to the cytoplasm [83]. The export from the nucleus is mediated by the chromosome-region maintenance 1 protein, CRM1 [31]. In the cytoplasm, semioxidized HMGB1 (Cys23-Cys45 disulphide and Cys106 thiol) leads to the activation of caspase-3 and caspase-9 and promotes the induction of the mitochondrial pathway of apoptosis. But it also binds to the protein beclin1 and favours the formation of the autophagosome [84]. Under proautophagic conditions beclin1 forms a complex with the proteins ambra1, VPS34, and VPS15 that initiates the formation of the phagophore [85]. The binding of HMGB1 to beclin1 favours autophagy and simultaneously inhibits apoptosis [27, 86]. Moreover, P53 is a negative regulator of HMGB1-beclin1 interactions in the cytoplasm, and loss of P53 increases interactions between HMGB1 and beclin1 [55].

HMGB1 also controls autophagy as a direct transcriptional regulator of the heat shock protein *β*1 (HSP*β*1), which is a regulator of actin cytoskeleton dynamics [86]. Therefore, the suppression of HSP*β*1 expression avoids the dynamics necessary to the progression of the autophagosome and consequently inhibits autophagy as well as mitophagy [86]. Class III phosphatidylinositol-3 kinase (PI3K III) activity is required for the activation of autophagy [87] and HMGB1 promotes the formation of the beclin1-PI3K III complex [88], which is necessary for triggering autophagosome nucleation [89], probably by mitogen-activated protein kinase kinase (MEK) and protein-serine/threonine kinases (ERK1/2) signalling [88].

## 6. Extracellular HMGB1 Functions and Effects on Other Cells That Contribute to Cancer Progression

**Figure 4:**
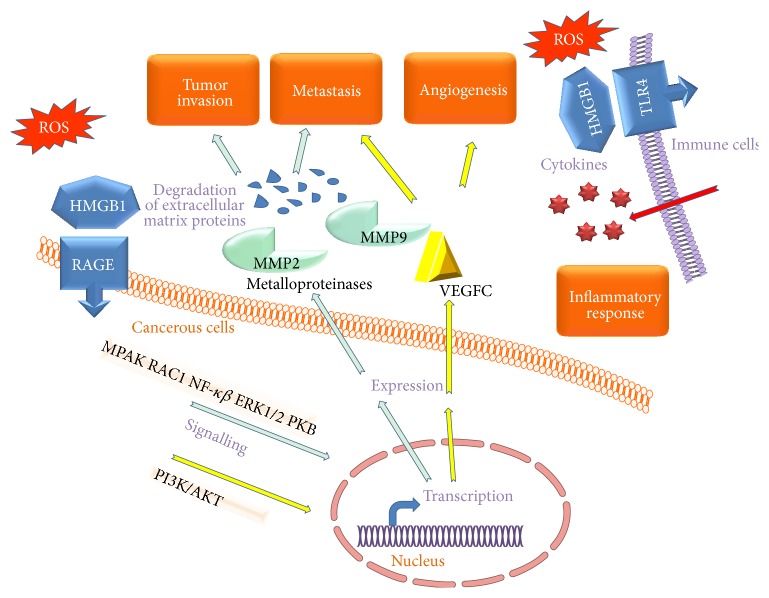
Simplified model of the effect caused by extracellular HMGB1, after oxidative stress, and upon the inflammatory response, invasion, metastasis, and angiogenesis.

If cancer cells do not cope with redox imbalance and undergone necrosis, the released HMGB1 induces diverse responses over the cells in the microenvironment (Figure 4), which contribute to tumour cell survival and the development of metastases [90]. These effects of extracellular HMGB1 are linked to poor prognosis in several cancers including prostate, colon, pancreas, and breast [80].

The extracellular HMGB1 binds to diverse receptors in several cells, alone or forming heterocomplexes with other immunogenic molecules. Reduced HMGB1 (three thiols in Cys23, Cys45, and Cys106) binds to RAGE and induces beclin1-dependent autophagy [84]. RAGE is expressed in macrophages, cancer cells, and cells in the microenvironment of tumours such as leukocytes, endothelial cells, and fibroblast [91]. Overexpression of RAGE and HMGB1 has been observed during cancer progression, invasion, and metastasis [92]. Conversely, blockade of RAGE-HMGB1 signalling suppresses tumour growth and metastases [93].

Semioxidized HMGB1 binds to TLR4 receptors in the immune cells and produces the release of cytokines, whereas reduced HMGB1 does not bind to TLR4. However, the reduced form binds to CXCR4 receptor forming a heteromer with the C-X-C motif chemokine 12 (CXCL12) and this interaction signals cell migration, thus promoting recruitment of motile inflammatory cells [94]. When all the thiol groups of HMGB1 have been oxidized to sulfonates, the molecule loses both the cytokine-inducing and chemoattractant activity [95]. In addition, HMGB1 forms complexes with other immune-stimulatory molecules as the lipopolysaccharide (LPS), the TLR2 ligand Pam3CSK4, nucleosomes, interleukin-1*β* (IL-1*β*), RNA, and DNA, which bind to diverse receptors in the cellular membrane or in the membrane of endosomes [37].

The migration of endothelial cells is necessary for angiogenesis and tumour growth and HMGB1 overexpression is associated with an increased angiogenic potential of the endothelial cells [96]. The molecules by which HMGB1 stimulates this proangiogenic response in the endothelial cells include targets of the vascular endothelial growth factor (VEGF) and platelet-derived growth factor (PDGF) as well as increased activity of matrix metalloproteinases, integrins, and NF-*κ*B [96].

## 7. The Function of HMGB Proteins and Other Redox Sensors during Oxidative Stress in Ovarian Cancer

Distinct cyto- and histopathology disorders in ovaries have been related to cancer malignancies and the epithelial origin (epithelial ovarian cancer or EOC) is the most frequent (80%) cause. There is some controversy about whether EOC is initiated in the ovarian surface epithelium or in the fallopian tube, since both share a common embryogenic origin [97].

OS has been proposed as a cause of ovarian cancer. ROS are generated during ovulation, and indeed several factors that reduce the number of ovulatory cycles along women life (oral contraceptive pills, pregnancies, and lactation) diminish the risk to have this type of cancer [98, 99]. Two hypotheses have been formulated to explain how the increase of ROS production accompanying ovulation might induce the carcinogenesis. In the “incessant ovulation” hypothesis, it is assumed that repeated cycles of apoptotic cell death and repair at the ovarian surface epithelium eventually generate OS and irreparable genetic damage; tumour suppressor genes become mutated and cells become malignant. The major epithelial origin of ovarian cancer could be a consequence of the less robust DNA repair mechanisms in the surface epithelial cells of the ovary [100]. In a second view, the “incessant menstruation” hypothesis, ROS are generated through the Fenton reaction supported by the iron present in heme released after lysis of red blood cells by macrophages [101].

Common gene mutations associated with OS, and which are found in the surface epithelial cells of the ovary, affect in 50-80% of ovarian cancers to the protein P53 and in 30% of ovarian cancers to RB. Other frequent mutations affect the small GTPases, RAS proteins, whose mutations produce resistance against OS-induced apoptosis, 8-oxoguanine DNA glycosylase (OGG1) whose mutation prevent the repair of oxidized guanine and increase C to T transitions, and the mutS homolog 2 (MSH2), involved in DNA mismatch repair [102, 103].

Enzymatic and nonenzymatic oxidative defence systems are necessary to cope with the oxidative environment that persists in the ovary. Among the enzymatic systems, superoxide dismutase, catalase, glutathione peroxidase, and glutathione reductase have been described in ovary [104]. The transcription factor NRF2 in healthy cells senses the redox state and activates the expression of genes related to protection against ROS damage through binding to AREs that are present in the promoters of the target genes. Although NRF2 is not a molecular redox sensor by itself, its translocation to the nucleus depends on the dissociation of its partner, the redox sensor KEAP1, which is E3 ligase adapter that in absence of ROS retains NRF2 in the cytoplasm and targets it for degradation in the proteasome [105–107]. NRF2 is also targeted for degradation in the proteasome by a KEAP1-independent mechanism that implies the phosphorylation of specific serines in the NEH6 domain of NRF2 by glycogen synthase kinase-3 (GSK3) and the interaction with the ubiquitin ligase adapter TrCP and the Cullin1/Ring-Box  1, E3 ubiquitin protein ligase (RBX1) complex [108]. OS affects the redox state of cysteine residues of KEAP1 and prevents NRF2 ubiquitination; in these conditions NRF2 enters the nucleus where it binds, together with the MAF proteins [109, 110], to AREs in the promoters of its target genes [111]. After restoration of the redox balance SRC-kinases will promote the export of NRF2 again to the cytoplasm for degradation [112]. The KEAP1-NRF2 pathway regulates both mitochondrial and cytosolic ROS production through NADPH oxidase [113]. Abnormal activation of NRF2 is a major event during ovarian carcinogenesis [22] and it is frequently due to RBX1 alterations [114]. A direct interaction between the two major redox sensors, KEAP1-NRF2 and HMGB1, which are implicated in the onset and progression of cancers related to OS, has not been reported; however they might converge in several signalling pathways. A cross talk between NRF2 and HMGB1 during the response to DNA damage has been proposed; it is thought that the NRF2-ARE pathway may regulate time kinetics of HMGB1 release; ROS and HMGB1 levels will then modulate the response to DNA damage [115].

OS activates the oncoprotein AKT in several cell types; the activation of serine/threonine kinase AKT is achieved either by a direct phosphorylation cascade or by inactivation of the phosphatase and tensin homolog protein PTEN [116]. Signalling pathways for AKT activation include those elicited by the EGF receptor, phosphatidylinositol-4,5-bisphosphate 3-kinase (PI3K), and integrins [117–119]. Activated AKT controls apoptosis and cellular proliferation and migration, as well as DNA repair [120]. However, active AKT also downregulates the antioxidant systems; this causes an increase in ROS generation that, in turn, stimulates AKT activation and produces further OS in a vicious cycle [121]. Activation of the PI3K/AKT pathway is indeed associated with 40% of human ovarian cancers in The Cancer Genome Atlas Network [102, 103, 122, 123]. A triple association of oxidative stress, AKT activation, and ovarian cancer has not yet been proved in humans, although it has been found in surface epithelial cells of mouse ovary [118]. It has been demonstrated that the extracellular signalling of HMGB1 through RAGE and TLR4 receptors activates the PI3K-AKT/ERK1/2 pathway and contributes to proliferation of lung cancer cells [124]. A connection between NFR2 and AKT has also been recently reported [108].

HMGB1 is considered a biomarker for ovarian cancer [38, 39] and increased levels of interleukin-8 protein (IL-8) and HMGB1 correlate with poor prognosis in prostate and ovarian cancer cells [125]. Targeting HMGB1 by RNA interference inhibits ovarian cancer growth and metastasis [126]. The relevance of HMGB1 is of particular importance to hormone-related cancers, including ovarian origin [40]. In this sense, the interaction between the estrogen receptor (ER) and the estrogen responsive element (ERE) in the promoters of target genes is markedly minor (60-fold) in nucleosome DNA compared to that in free DNA and diverse approaches have shown that HMGB1 restructures the canonical nucleosome to facilitate strong ER binding [40]. Lymph node is a probable channel by which ovarian cancer cells may spread and invade other tissues. In human epithelial ovarian cancer, the protein HMGB1, together with tumour-associated macrophages, enhances lymphangiogenesis [127].

HMGB2 is also deregulated in EOC [128]. HMGB2 is part of the SET complex, which is composed of NM23, P32, SET, HMGB2, and APE1. This complex is also implicated in apoptosis and response to OS and DNA repair [128]. Tumours expressing low levels of SET, but high levels of NM23, or, alternatively, low levels of APE1, but high levels of HMGB2, have a better prognosis compared to other tumours [128]. Although the mechanisms producing these patterns are still unknown, the authors postulated that specific combinations of markers from the SET complex could be useful to classify patients for treatment [128].

## 8. Oxidative Stress in Prostate Cancer and the Function of HMGB Proteins and Other Redox Sensors

The human prostate anatomy displays a zonal architecture, corresponding to central, periurethral transition, peripheral zone, and anterior fibromuscular stroma. The majority of prostate carcinomas are derived from the peripheral zone, while benign prostatic hyperplasia arises from the transition zone [129]. Prostate contains a pseudostratified epithelium formed by three cell types: luminal, basal, and neuroendocrine [130]. However, a histopathological classification of prostate cancer subtypes, which differ in their prognosis or treatment, has not been possible. The majority of the diagnosed prostate cancers correspond to acinar adenocarcinomas that originate in the prostate gland and express the androgen receptor [129].

Increased ROS production in prostate cancer cells has been linked to diverse processes. The first one is the change observed in mitochondrial function. Frequently, the mitochondrial DNA isolated from prostate cancer cells contains an increased rate of mutations [131], which compromise the stability of the genome and the mitochondrial function, thus increasing ROS production. Upregulation of members of the membrane-bound NADPH oxidase protein complex (NOX1-5 and DUOX), which catalyses the production of superoxide from oxygen using NADPH as a cofactor [132], is another important source of intracellular ROS production. In human prostate cancer cells the levels of NOX2, NOX4, and NOX5 are increased [133]. As an additional source during prostaglandin biosynthesis, the catalytic activities of the cyclooxygenase enzymes (COXs) also produce ROS. The COXs proteins are present in two isoforms, COX1, constitutively and ubiquitously expressed, and COX2 that is overexpressed in cancerous prostate tissues [134]. Androgens, which are very important in prostate cancer development, also contribute to increasing ROS levels by signalling the transcription factor JUND [135] and the mitochondrial redox regulator P66SHC, a 66 kDa SRC homologous-collagen homologue (SHC) adaptor protein [136]. However, ROS levels could also be increased due to androgen deprivation [137, 138]. These results indicate that physiological levels of androgens are necessary to maintain the cellular redox equilibrium, and deviations caused by high or low production cause OS. Chronic inflammation, proliferative inflammatory atrophy (PIA), and infectious prostatitis constitute a prior stage to prostate malignancy [139, 140] and, in these conditions, activated inflammatory cells and secreted inflammatory cytokines contribute to ROS generation and therefore to carcinogenesis [139, 141].

Antioxidant defences are diminished in prostate cancer cells, oppositely to what could be expected taking into account the increased production of ROS. Superoxide dismutase (SOD1, SOD2) and catalase activities are downregulated [142, 143] and the master redox regulator NRF2 is significantly downregulated in human prostate cancer [21]. As a consequence of higher levels of ROS production and diminished antioxidant defences, several indicators of oxidative damage have been found and tested as diagnosis and prognosis markers in prostate cancer. These include increased F2-isoprostane [144] or 8-hydroxydeoxyguanosine [145] in urine and increased peroxide levels [137] or decreased levels of the antioxidant *α*-tocopherol [146] in serum.

Recently, functional links between OS and prostate cancer have been reviewed [138]. Oxidative damage and DNA damage, which may produce changes favouring the invasive behaviour of epithelial cells, have been described [147] as well as the shortening of telomeres, which may lead to chromosomal instability [148]. The levels of the tumour suppressor homeobox protein NKX3.1 are diminished in nearly all prostate cancers and metastases studied [149]; it has been suggested that NKX3.1 has a protective role against DNA damage [150]. This protein also links OS with prostate cancer in animal models; mutation of the homologous protein in mice displays deregulated expression of several antioxidant and prooxidant enzymes; in this model, progression to prostate adenocarcinoma is correlated with decreased superoxide dismutase activity and accumulation of oxidative damage in DNA and proteins [151].

Diverse cellular signalling pathways have been reported to play significant roles in the progression of prostate cancer [152]. Among them those regulated by the androgen receptor (AR) [153–155], estrogen receptors [156], PI3K/Akt/mTOR [157, 158], PTEN [159], NF-*κ*B [160], the epidermal growth factor receptor EGFR [161], and PDGF [162]. Also, ROS-activated matrix metalloproteinases, which promote invasion and metastasis, are activated in prostate cancer cells [133]. RND3, which contributes to cell migration, is also deregulated in prostate cancer [76]. Finally, it has been suggested that, during prostate cancer progression, genes expressed in embryonic developmental programs are reactivated [163]. In particular, elevated canonical Wnt signalling may play a role in the emergence of castration resistance [164, 165]. Activation of Hedgehog signalling [166, 167] and Notch [168] and fibroblast growth factor (FGF) signalling [169, 170] may also play significant roles in prostate cancer.

There are many interconnections between these signalling pathways. For instance, PTEN functions as a tumour suppressor by negatively regulating the PI3K/AKT signalling and, in 30–50% of prostate cancer cases, loss of PTEN function causes PI3K/AKT signalling upregulation [158]. In an early step of prostate carcinogenesis, PTEN undergoes copy number loss and this event is correlated with progression of prostate cancer to a more aggressive, castration-resistant, stage that does not respond to hormone therapy [171]. The upregulation of AKT/mTOR signalling pathway in prostate cancer occurs primarily through activation of AKT1 [172]. The consequences of AKT activation are mediated in part by activation of NF-*κ*B signalling via stimulation of inhibitor NF-*κ*B kinase, IKK [173]. The stimulation of AR signalling leads to activation of SRC oncogenic kinases that phosphorylate AR in prostate cancer cells and cause castration resistance and cellular proliferation and invasiveness [174]. PI3K/AKT signalling [175] and AR signalling [155] increase SKP2 abundance in prostate cancer cells. SKP2 is the S-phase kinase associated protein 2 involved in cell cycle progression; it is the component of the SCF complex that confers substrate specificity to E3 ligase for ubiquitination of many targets that are tumour suppressors, which are marked for degradation in the proteasome [176]. Remarkably, as explained along the review in precedent sections, several among these signalling pathways are elicited by the redox sensor NFR2 or by the HMGB proteins.

Finally, several research lines outline the direct importance of HMGB proteins in prostate cancer and their implications in therapy. Increased HMGB2 expression [177], HMGB1 expression [41], or coexpression of RAGE and HMGB1 [178, 179] has been associated with prostate cancer progression and has been correlated to poor patient outcome. Consequently, silencing of* HMGB1* [180] or* RAGE* [181] genes in prostate cancer cells resulted in decreased cellular viability.

## 9. Cisplatin, Chemoresistance, Oxidative Stress, and HMGB Proteins

Cisplatin (cis-diamminedichloroplatinum(II)) is commonly used in prostate, ovarian, and other cancers therapy. It binds to DNA and forms majorly intrastrand cross-links with guanines. This produces cytotoxicity by inducing a DNA damage stress response [182, 183]. Emodin, an effective ROS generator, in cotreatment with cisplatin remarkably enhances chemosensitivity in prostate cancer cells, compared with cisplatin alone [184]. Cisplatin also generates OS response in the cells [185] that, together with the OS response generated as a consequence of cancer disease, might affect the functions of HMGB proteins. Steroid hormones that induce HMGB1 overexpression sensitize cancer cells to cisplatin and carboplatin [186]. In the LNCaP prostate cancer cell line, combined treatment with estrogen and cisplatin increases HMGB1 expression and apoptosis more than cisplatin alone and this effect is mediated by interaction between estrogen and ER-alpha [187].

Indeed, cisplatin and HMGBs proteins are functionally related, since these proteins bind with higher affinity to platinated DNA than to unmodified DNA [188]. The reduced (three-thiol) form of HMGB1 has a higher affinity for platinated DNA than the semioxidized form [189]. In this sense, the success of cisplatin chemotherapy toward testicular tumours has been attributed to the specific expression in testis of HMGB4 that lacks one of the cysteine residues that forms the disulphide bond in the other HMGB proteins [30].

The initial positive response to cisplatin treatment is frequently limited by development of broad resistance against radio- and chemotherapies. Therefore, there is much interest in understanding the mechanisms responsible for development of resistance in the treatment of ovarian and prostate cancers and other types of cancers. The proteins HMGB1, HMGB2, HSC70, GRP58, and GAPD form a nuclear complex, which alters DNA conformation, and they have been associated* in vivo* with resistance to chemotherapeutic drugs in ovarian cancer patients [190]. In an ovarian cell line resistant to platinum-treatment some genes were overexpressed including those encoding for matrix metalloproteinases (MMP3 and MMP12) and HMGB2, while genes that encode for extracellular matrix proteins were downregulated as well as genes involved in the regulation of cell cycle and growth [191]. In a wide-genome study of genes associated with platinum-based chemotherapy resistance in ovarian cancer, several connections with the OS response have been found; these include the response mediated by NRF2, P53, and TGF*β* signalling [192, 193], which have many links to HMGB proteins as already explained. Nucleus accumbens-1 (NAC1), a nuclear factor belonging to the BTB/POZ gene family, also modulates sensitivity of ovarian cancer cells to cisplatin by altering the HMGB1-mediated autophagic response [194].

Clusterin, a chaperone protein upexpressed in prostate cancer, stabilizes Ku70/BAX complexes, sequestering BAX from its ability to induce mitochondrial release of cytochrome *c*, thus avoiding subsequent apoptosis and promoting resistance to cisplatin; the secreted clusterin form is expressed in aggressive late stage tumours, and although its high expression may be considered an adaptive response to OS, it enhances the survival potential of cancerous cells [195]. Overexpression of riboflavin kinase, necessary for synthesis of FAD and glutathione reduction, is upregulated in cisplatin-resistant cells and it is related to prostate cancer progression [196]. The ubiquitin-specific protease 2a (USP2A), a deubiquitinating enzyme overexpressed in prostate adenocarcinomas, confers resistance to cisplatin; USP2A increases intracellular reduced glutathione content, reduces ROS production, and impairs the activation of apoptosis [197].

Resistance to cisplatin has been also attributed to DNA repair enzymes, which are able to remove lesions caused by cisplatin on DNA [182]. The mechanism of DNA repair is however inhibited by HMGB proteins that contribute to cytotoxicity both* in vitro* [198–200] and* in vivo* assays [201].

## 10. Conclusions and Perspectives

ROS overproduction and imbalance are a primary cause of malignancy in the onset of cancer. Cells have evolved multiple strategies in response to ROS production and HMGB proteins play a major role in many molecular mechanisms participating in these responses. In the nucleus, HMGB proteins affect DNA repair, transcription, and chromosomal stability; in cytoplasm they determine key decisions that finally lead towards autophagy or apoptosis; as extracellular signals they produce changes that affect the microenvironment of the tumour and attract cells from the immune system. In turn, the inflammatory onset can increase ROS production and therefore enhances the response. HMGB1 and HMGB2 are expressed at the highest levels in immune cells and, besides, they have been related to cancers, which are hormone-responsive, such as ovarian and prostate cancers. Since HMGB proteins have many different functions and are necessary in healthy cells, an improved strategy to modulate their role in cancer progression could be to act through other proteins interacting specifically with them. The identification of HMGB partners, which could be univocally associated with specific cancerous processes or with mechanism of cisplatin resistance, is a field of interest for ongoing translational cancer research. Interactome strategies are outstanding for the development of these research lines.
